# Integrative taxonomy suggests hidden diversity within the fish genus *Cyttopsis* (Zeiformes, Parazenidae)

**DOI:** 10.1111/jfb.70190

**Published:** 2025-08-19

**Authors:** Rafael Bañón, Francisco Baldó, Juan Carlos Arronte, Alejandro de Carlos, José‐ Daniel Barreiro‐Vázquez, Ángel Sebastián Comesaña, David Barros‐García

**Affiliations:** ^1^ Grupo de Estudo do Medio Mariño Ribeira Spain; ^2^ Centro Oceanográfico de Cádiz (COCAD‐IEO) CSIC Cádiz Spain; ^3^ CIIMAR/CIMAR LA, Interdisciplinary Centre of Marine and Environmental Research University of Porto, Terminal de Cruzeiros do Porto de Leixões Matosinhos Portugal; ^4^ Centro Oceanográfico de Santander (COST‐IEO) CSIC Santander Spain; ^5^ Departamento de Bioquímica, Xenética e Inmunoloxía, Facultade de Bioloxía Universidade de Vigo Vigo Spain; ^6^ Centro de Investigación Mariña Universidade de Vigo Vigo Spain; ^7^ Departamento de Anatomía, Produción Animal e Ciencias Clínicas Veterinarias, Hospital Veterinario Universitario Rof Codina, Facultade de Veterinaria Universidade de Santiago de Compostela Lugo Spain; ^8^ Centro de Apoyo Científico y Tecnológico a la Investigación Universidade de Vigo Vigo Spain

**Keywords:** Atlantic Ocean, cryptic species, *Cyttopsis cypho*, *Cyttopsis rosea*, morphology, species delimitation

## Abstract

Nine specimens of *Cyttopsis rosea* (Zeiformes: Parazenidae) were collected during scientific surveys at three different locations in the northeast Atlantic. All nine specimens were included in the molecular analysis, adding new cytochrome c oxidase subunit I sequences to public databases. Of these, six specimens were retained for detailed morphological examination. Morphological measurements and counts agree with previous descriptions and confirm the identification of *C. rosea*. However, molecular species delimitation analyses suggested cryptic diversity, identifying three molecular operational taxonomic units, contrary to the current status of a single species with a worldwide distribution. The integration of classical and molecular taxonomy proved essential for accurately delimiting and characterizing this species, enhancing our understanding of its intraspecific morphological and molecular variability. A literature review of the available morphological data of *Cyttopsis rosea* between Atlantic and Pacific specimens showed differences in the caudal peduncle length, number of vertebrae and the number of scales in the lateral line, which could support the existence of different species. Based on the resulting data and the available literature, an updated key of the accepted members of the family Parazenidae is provided.

## 1 INTRODUCTION

Zeiformes is a primarily benthopelagic order of acanthomorph fishes that live and feed near the seafloor at depths of 50–1000 m. They are distributed globally in tropical and temperate regions, with some species having near global ranges, while others are regional endemics (Peters et al., 2024). The family Parazenidae comprises three genera and four species of zeiform fishes distributed worldwide in tropical and temperate waters on the continental shelf and slope of the Atlantic, Pacific and Indian Oceans. The family was reviewed by Heemstra (1980) and later by Tyler et al. (2003) and Grande et al. (2018) based on molecular and morphological characters, confirming the monophyly of the family. The parazenid genus *Cyttopsis* Gill, 1862 contains two valid species, the Rosy dory *Cyttopsis rosea* (Lowe, 1843), found in the Atlantic and Indo‐West Pacific, and the little dory *Cyttopsis cypho* (Fowler, 1934), which is restricted to the eastern Indian and western Pacific Oceans (Mizumachi et al., 2022).

*Cyttopsis rosea* is a demersal species found between 150 and 962 m depth (Bañón et al., 1997; Maurin & Quéro, 1981). It was originally described by Lowe (1843) as *Zeus roseus* on the basis of two specimens from the Atlantic Madeira Islands. It is currently distributed worldwide, occurring in the western Atlantic from Venezuela to Canada, in the eastern Atlantic from the British Isles to Gabon, in the western Indian Ocean from Somalia to South Africa (KwaZulu‐Natal), the Maldives and India, and in the western Pacific off Japan, eastern and western Australia, New Caledonia and New Zealand (Heemstra, 2022; Munroe et al., 2015). *Cyttopsis itea* Jordan & Fowler, 1902 and *Paracyttopsis scutatus* Gilchrist & von Bonde, 1924 are considered synonyms of *C. rosea* (Heemstra, 1980).

In the eastern Atlantic, *C. rosea* has been found to be expanding its range northwards into European Atlantic waters as a result of warming waters associated with climate change and the tropicalization phenomenon. It was reported in Portugal in 1963 and has expanded northwards to 43°N in 1968, 44°N in 1969, 49°N in 1987 and 52°N in 1988 (Quéro et al., 2023). According to the Red List of the International Union for Conservation of Nature (IUCN), *C. rosea* is considered globally to be of Least Concern (Munroe et al., 2015).

Molecular taxonomy, including the analysis of DNA sequence data, provides a strong assessment of genetic relationships and has revealed inconsistencies in taxa defined by traditional phenotypic methods. The analysis of DNA barcode sequences using various clustering techniques offers an efficient method for recognizing putative species (operational taxonomic units) (Kekkonen & Hebert, 2014). Integrated morphological and molecular analyses allow more accurate identification of fish species, facilitating the description of new species and the discovery of synonymous or cryptic species (Bañón et al., 2024). Molecular tools have been successfully used to highlight some of these findings in zeiform fishes (Costa et al., 2012; Kai & Tashiro, 2019; Ward et al., 2008).

The aim of this research was to add new morphological and molecular data from *C. rosea* caught in northeast Atlantic waters to the global dataset, and to review the available taxonomic and biogeographical knowledge to document the possible occurrence of cryptic diversity.

## 2 MATERIALS AND METHODS

### 2.1 Ethics

The species described in this paper are not listed as threatened on either the IUCN Red List or any national, regional or local schedules of protected fauna. All required permits for collection were obtained from the Instituto Español de Oceanografía (IEO), Consejo Superior de Investigaciones Científicas (CSIC).

### 2.2 Specimen collection and processing

**FIGURE 1:**
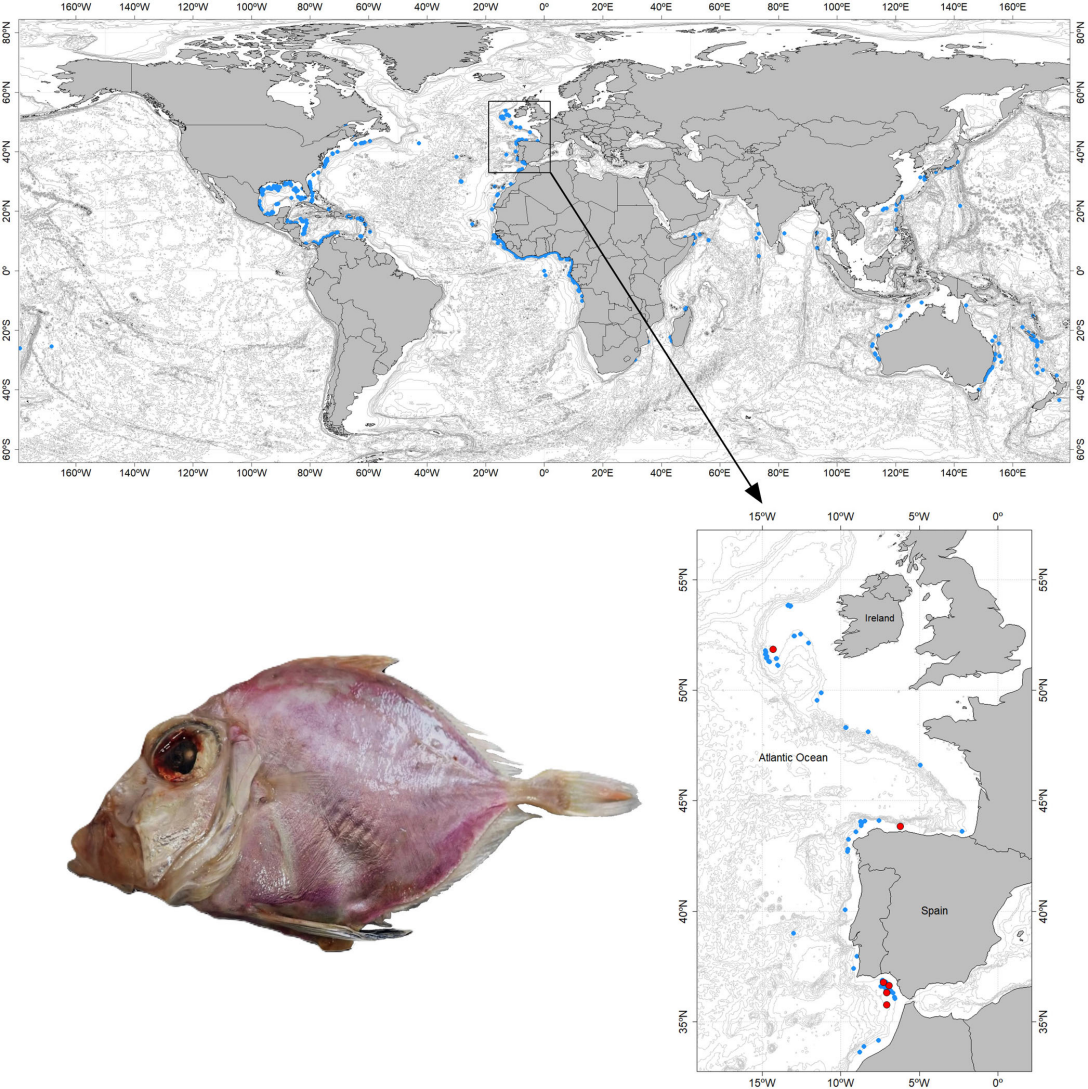
Sampling location of *Cyttopsis rosea* specimens (red dots). The global distribution of the species is also shown (GBIF.org, 2025; OBIS, 2025), including historical records from the Spanish research bottom trawl surveys in the Porcupine Bank, the north coast of Spain and the Gulf of Cadiz (light blue dots).

Nine specimens of *C. roseus* were collected through trawling during several research surveys conducted in various areas of the North Atlantic Ocean (Figure 1): (i) Porcupine 2022, conducted off the west coast of Ireland; (ii) Demersales 2022, in Galician waters and the Cantabrian Sea, and (iii) ARSA 2022 and ARSA 2024, conducted in the Gulf of Cadiz (southern Spain). Specimens were preliminarily identified on board, and entire specimens or tissues were preserved frozen (−28°C). Following tissue extraction for molecular analysis, six voucher specimens were preserved frozen and deposited in the fish collection at the Museo Luis Iglesias de Ciencias Naturais in Santiago de Compostela (MHN USC), under reference numbers MHN USC 25228‐1 to 25228‐6. Additional tissue samples from three specimens collected in the Gulf of Cadiz were used for DNA species delimitation analyses and are deposited at the Universidade de Vigo.

### 2.3 Morphological analyses

Morphological analysis was carried out according to Mizumachi et al. (2022). Measurements were taken to the nearest 0.1 mm, using a digital calliper, except for total length (*L*_T_) and standard length (*L*_S_), which were measured to the lower millimetre using an ichthyometer. A comprehensive review of morphological characters reported in the ichthyological literature was compiled to search for geographic differences in the species. Vertebrate counts were determined from radiographs taken using mammography computed radiography equipment (Fujifilm Capsula XL II) with exposure factors of 44 kVp × 35 mAs.

### 2.4 DNA extraction, PCR amplification and sequencing

DNA extraction was performed from muscle tissue samples preserved in 70% ethanol at −20°C using the E.Z.N.A. Tissue DNA Kit (Omega Bio‐Tek) according to the manufacturer's instructions. The DNA barcode region of the mitochondrial gene encoding the enzyme cytochrome c oxidase I was amplified using the polymerase chain reaction. For this purpose, the Horse‐Power Taq DNA Polymerase Master Mix (Canvax) was employed in combination with the C_FishF1t1‐C_FishR1t1 universal primer cocktail for fish DNA barcoding (Ivanova et al., 2007). The temperature regime was as follows: 98°C for 5 min, 35 cycles of 94°C for 35 s, 52°C for 35 s and 72°C for 50 s, and 72°C for 7 min. Sanger cycling sequencing reactions were performed with BigDye Terminator v3.1, and the resulting products resolved in a SeqStudio Genetic Analyser at the Centro de Apoyo Científico y Tecnológico a la Investigación facilities (University of Vigo). Trace files were interpreted and DNA alignment was conducted using MEGA version 11 (Tamura et al., 2021).

DNA sequences, specimen photographs and associated metadata have been deposited in the Barcode of Life Database (BOLD Systems; www.boldsystems.org) as part of the projects titled ‘Porcupine Bank Fish’ (code PORF, process ID: PORF357‐25) and ‘Unusual Atlantic Fishes’ (code UNAFI, process ID UNAFI 014‐25 to UNAFI 021‐25). Additionally, the barcode sequences have been submitted to GenBank (https://www.ncbi.nlm.nih.gov/genbank/) under accession numbers PV009291‐PV009299.

### 2.5 Phylogenetic analyses

Publicly available cytochrome c oxidase subunit I (*COI*) sequences previously assigned to the family Parazenidae were retrieved from BOLD‐Systems and GenBank repositories (February 2025). After validation, the final dataset comprised 54 *COI* sequences belonging to family Parazenidae plus one outgroup species (Piraten perch *Aphredoderus sayanus* [Gilliams, 1824]). To apply delineation methods based on phylogenetic tree topologies, trees were constructed using both Bayesian inference for generalized mixed yule coalescent (GMYC) and maximum likelihood (ML) for Bayesian Poisson tree processes (bPTPs). To this end, an optimal partition strategy and a substitution model were determined using PartitionFinder v1.1.1 (Lanfear et al., 2012) and jModelTest2 (Darriba et al., 2012; Guindon & Gascuel, 2003), respectively. Single partition and general time reversible (GTR) with invariant sites (+I) were therefore selected for further analyses. BEAST V2.5.2 was used for Bayesian inference, with two runs of 10 million generations with four independent chains and sampling every 1000 generations. Convergence of the analyses was addressed with TRACER.1.7.0 (Rambaut et al., 2018). The final consensus tree was obtained using a posterior probability of 0.9, discarding the first 25% of sampled trees, and generated in TreeAnnotator v2.4.5. The tree was then visualized with FigTree v.1.4.3 (http://tree.bio.ed.ac.uk/software/figtree/).

The ML analysis was conducted through the CIPRES portal using the IQ‐tree tool on XSEDE (version 2.4.0), with 1000 rapid bootstrap replicates to provide statistical support for the nodes (Miller et al., 2010). For the PTP analysis, the tree with the highest likelihood from the ML analysis was selected as the input.

### 2.6 Species delimitation

Several DNA delimitation analyses were conducted to assess the taxonomic status of the family Parazenidae. First, the distance‐based Barcode Index Number (BIN) was retrieved from BOLD Systems (https://www.boldsystems.org/) (Ratnasingham & Hebert, 2013). The second distance‐based method applied was the Assemble Species by Automatic Partitioning (ASAP) (Puillandre et al., 2021), which is available via a web server (https://bioinfo.mnhn.fr/abi/public/asap/). In this method, p‐distance was calculated for the *COI* alignment without an outgroup, and the optimal partition was selected based on the ASAP score and threshold distance.

Four tree‐based delimitation approaches were used for analysis. First, two modifications of the PTP algorithm (Zhang et al., 2013) were applied: bPTP, which incorporates Bayesian support values to the delimited species, and mPTP, which uses a multi‐rate criterion to account for varying levels of intraspecific genetic diversity (Kapli et al., 2017). These analyses were conducted on web servers: bPTP was run at (https://species.h-its.org/ptp/) and mPTP at (https://mptp.h-its.org/#/tree). In both cases, the ML tree was used as input, after removing the outgroup, to achieve optimized results (Zhang et al., 2013).

Next, the GMYC model was applied, which identifies the time threshold defined by coalescent processes on ultrametric trees (Fujisawa & Barraclough, 2013; Pons et al., 2006). A variation of this model, the multi‐rate GMYC (mGMYC), was also used, which assumes multiple independent coalescent processes across the tree's topology (Monaghan et al., 2009). Both analyses were performed using the R package ‘splits,’ with the ‘gmyc’ function, specifying ‘method = single’ for GMYC and ‘method = multiple’ for mGMYC (Fujisawa & Barraclough, 2013).

## 3 RESULTS

### 3.1 Systematic account

#### 3.1.1 *Cyttopsis rosea* (Lowe, 1843)

*Zeus roseus* Lowe, 1843: 85, off Madeira, northeastern Atlantic, Syntypes: (2) BMNH 1852.9.13.104; *Cyttus roseus* Günther, 1860: 396 (description); *Cyttus roseus* Vaillant, 1888: 349 (description); *Cyttopsis roseus* Goode & Bean, 1895: 227 (description); *Cyttus (Cyttopsis) roseus* Cadenat, 1953: 1079 (description); *Cyttus roseus* Poll, 1954: 19 (description); *Cyttus roseus* Furnestin et al., 1958: 427 (description); *Cyttopsis roseus* Heemstra, 1980: 5 (description); *Cyttopsis roseus* Heemstra, 1986: 436 (description. key); *Cyttopsis rosea* Roa‐Varón et al., 2003: 11 (description); Heemstra, 2016: 2227 (description, key).

#### 3.1.2 Material examined

MHN USC 25228‐1, 137 mm TL, 9 October 2022, Cantabrian Sea; 43.843°N, 6.211°W, 407 m depth; MHN USC 25228‐2, 136 mm TL, 9 October 2022, Cantabrian Sea; 43.843°N, 6.211°W, 407 m depth; MHN USC 25228‐3, 77 mm TL, 10 November 2024, Gulf of Cádiz, south Spain; 36.321°N, 7.071°W, 519 m depth; MHN USC 25228‐4, 135 mm TL, 13 September 2024, Porcupine Bank, western Ireland; 51.855°N, 14.309°W, 356 m depth. MHN USC 25228‐5, 91 mm TL, 5 November 2022, Gulf of Cadiz, south Spain; 36.638°N, 6.943°W, 409 m depth. MHN USC 25228‐6, 150 mm TL, 6 November 2022, Gulf of Cadiz, south Spain; 36.795°N, 7.278°W, 475 m depth.

#### 3.1.3 Description

**FIGURE 2:**
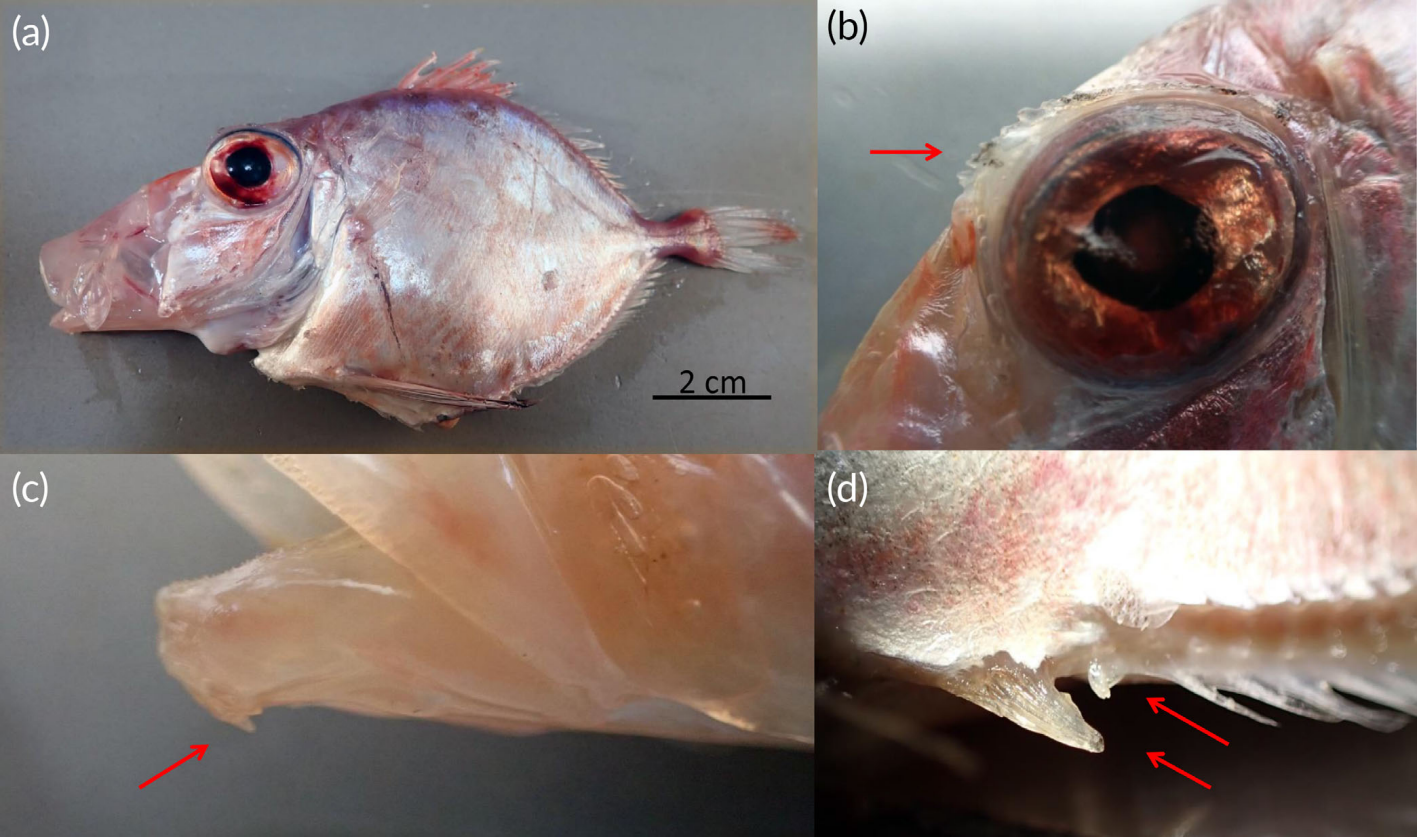
*Cyttopsis rosea* from the northeast Atlantic. Arrows show the described character: (a) whole specimen MHN USC 25228‐4, 135 mm *L*_T_ showing the protusible mouth, (b) detail of eye with supraorbital crest, (c) small spine on the lower jaw and (d) anal fin with two anterior spines.

**FIGURE 3:**
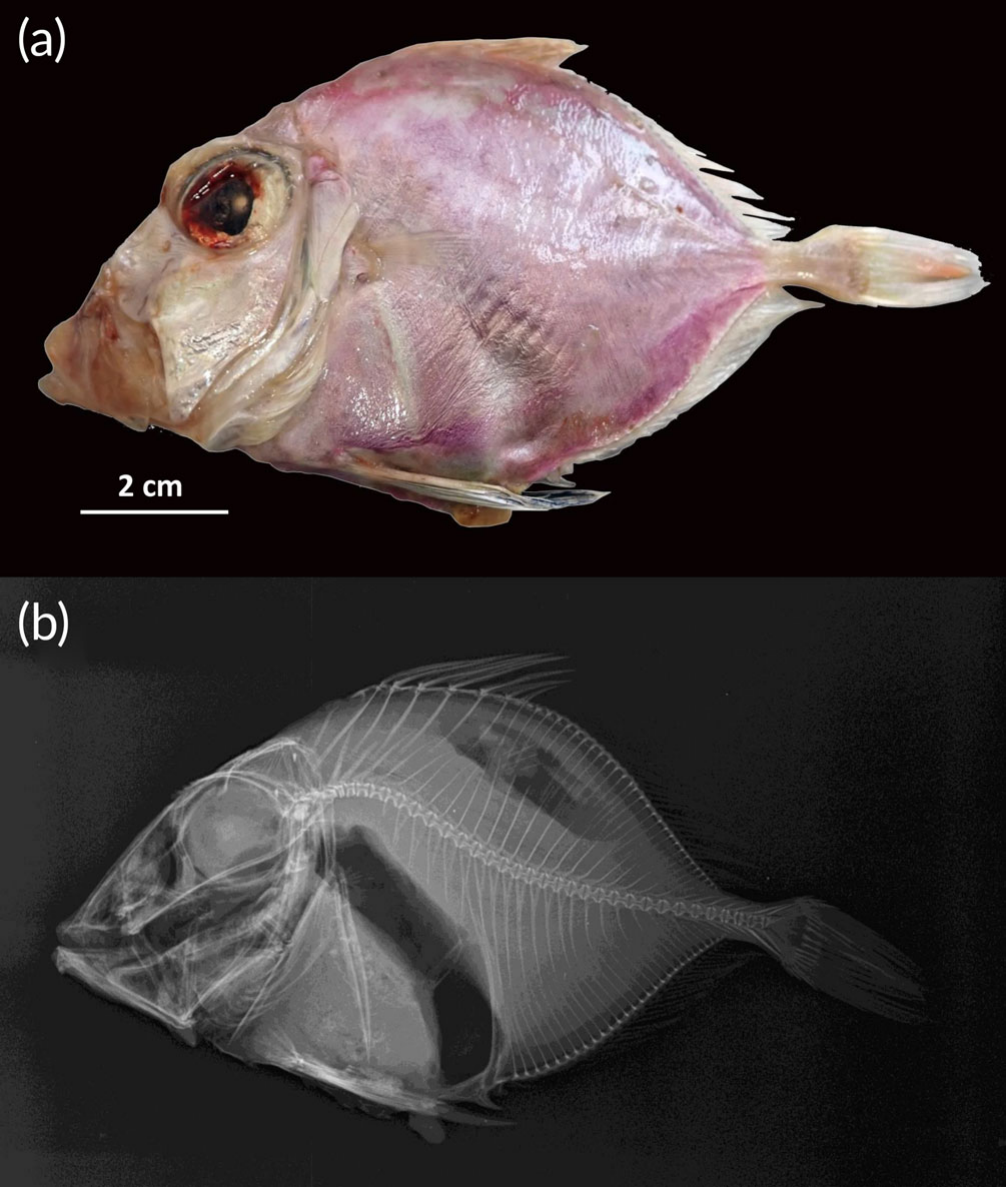
Specimen of *Cyttopsis rosea* Museo Luis Iglesias de Ciencias Naturais in Santiago de Compostela MHN USC 25228‐6, 150 mm *L*_T_, caught in the Bay of Cádiz (northeast Atlantic): (a) lateral view of the specimen and (b) radiograph of the specimen.

Body oval and compressed (Figures 2a and 3), body depth greater than head length and contained 1.7–1.9 times in the standard length; head large, contained 2.3 to 2.6 times in the standard length; snout slightly longer than the eye diameter; eye very large, its diameter 2.5 to 3 times in the head; edge of bony orbit with small spines anteriorly (Figure 2b); mouth large, oblique with a protruding upper jaw; with dentaries with two small spines at the symphysis below (Figure 2c); interorbital width 1.5–1.9 in eye diameter; pectoral fin inserted before the spinous dorsal fin, its length much shorter than head length; pelvic fin long, inserted nearly in the vertical from the insertion of the pectorals, its length 1–1.3 times in the head length; first anal spine flattened and immovable (Figure 2d); ventral midline between pelvic and anal fins broad, flattened, with large spiny bucklers; a row of low bony ridges on each side at the base of soft dorsal and anal fins; 66–72 scales in lateral line; caudal peduncle length 4.9–5.8 times in the head length, number of vertebrae 32. The key morphometric and meristic characteristics are provided in Table 1. A revision of the morphological characters documented in the ichthyological literature for the Atlantic and Pacific specimens is presented in Table 2.

**TABLE 1:** Measurements and counts of specimens of *Cyttopsis rosea* MHN USC 25228‐1 to MHN USC 25228‐6.

Character	1	2	3	4	5	6
Total length (mm)	137	136	77	135	91	150
Standard length (mm)	114	113	63	112	75	124
As % *L*_S_						
Head length	38.2	42.5	44.4	39.3	42.4	41.5
Snout length	16.3	19.5	18.6	17.2	19.3	19.8
Postorbital length	6.6	7.5	8.3	6.5	7.6	7.8
Eye diameter	15.4	15.9	15.9	15.2	14.1	14
Interorbital space	8.3	8.6	8.4	8.6	8	9.1
Predorsal length	52.3	55.0	55.2	52.3	56.7	54
Dorsal base fin length	52.2	52.7	47.3	52.3	49.7	53.2
Preanal length	58.8	61.2	54.0	59.8	57.9	60.1
Anal base fin length	42.6	38.5	34.1	40.8	38.4	40.7
Prepectoral length	38.0	39.6	40.5	42.3	41.1	37.5
Preventral length	40.4	39.2	36.8	35.5	41.3	38.5
Pelvic‐anal distance (LPA2)	14.0	17.7	16.7	16.3	19.2	18.9
Pectoral‐anal distance (LPA1)	31.1	30.1	30.6	35.0	33.3	28.9
Pectoral‐pelvic (LPP)	20.2	20.1	19.0	19.6	19.1	18.5
Dorsal‐pectoral (LDP1)	26.3	26.1	23.8	24.4	26	26.6
Dorsal‐pelvic (LDP2)	49.4	50.6	46.3	51.2	50.7	45.8
Pectoral fin length	13.4	13.5	13.0	14.1	14.4	13.9
Pelvic fin length	34.5	37.2	46.2	34.1	40.9	31.2
Body depth	56.1	56.2	53.8	58.7	56.3	54.7
Upper jaw length	18.4	17.7	19.0	18.8	20	18.5
Lower jaw length	24.6	22.6	23.0	22.7	22.9	23.1
Caudal peduncle minimum height	5.7	5.3	6.0	5.5	6	5.2
Caudal peduncle length	6.6	7.7	9.0	7.6	7.7	8.3
Length 3° dorsal spine (longest)	17.5	18.9	14.4	15.2	16	16.1
Length 1° anal spine	3.9	4.6	2.7	3.3	3.7	3.5
Distance between pelvic fin bases	10.2	11.3	12.9	12.5	11.5	10.3
Meristic						
Dorsal fin rays	VII + 27	VIII+27	VII + 29	VII + 28	VII + 29	VII + 29
Anal fin rays	I + 30	II + 30	I + 30	II + 29	I + 30	II + 29
Pectoral fin rays	14	14	14	14	14	14
Ventral fin rays	9	9	9	9	9	9
Developed gillraker	1 + 10	2 + 10	1 + 9	2 + 10	2 + 9	2 + 8
Branchiostegal rays	7	7	7	7	7	7
Vertebrae	32	32	32	32	32	32
Scales in lateral line	66	72	69	70	69	70
Soft dorsal‐fin base scutes	27	25	28	26	27	28
Soft anal‐fin base scutes	28	30	27	27	28	27
Caudal fin rays	4 + 13 + 4	4 + 13 + 4	3 + 13 + 4	3 + 13 + 3	3 + 13 + 4	3 + 13 + 3

**TABLE 2:** Differences in meristic and morphometric characters in *Cyttopsis rosea* specimens between the Atlantic (Lowe, 1843; Cadenat, 1953; Poll, 1954; Furnestin et al., 1958; Bañón et al., 1997; this manuscript) and Pacific (Mizumachi et al., 2022).

Character	Atlantic	Pacific
Total length (mm)		
Standard length (mm)	63–124	56.5–186.6
As % *L*_S_		
Head length	37–44.4	38.8–47.6
Snout length	16.3–25.6	17.8–23.3
Postorbital length	6.5–8.3	–
Eye diameter	13–15.9	14.7–19.1
Interorbital space	6.6–9.1	5.8–8.0
Predorsal length	52.3–59.5	50.4–59.8
Dorsal base fin length	47.3–53.2	48.2–55.4
Preanal length	50.8–67.8	50.3–61.5
Anal base fin length	34.1–42.6	36.5–42
Prepectoral length	37.5–42.3	–
Preventral length	35.5–41.3	–
Pelvic‐anal distance (LPA2)	14–19.2	11–23.7
Pectoral‐anal distance (LPA1)	28.9–35	24.6–33.5
Pectoral‐pelvic distance (LPP)	18.5–20.2	19.3–26.4
Dorsal‐pectoral distance (LDP1)	23.8–26.6	23.4–29.2
Dorsal‐pelvic distance (LDP2)	45.8–51.2	44.7–58
Pectoral fin length	13–14.4	12.4–17.6
Pelvic fin length	31.2–46.2	28.4–48.8
Body depth	47.4–60.7	47.7–60.8
Upper jaw length	17.7–20.5	16.4–22.7
Lower jaw length	22.6–24.6	22.1–26.9
Caudal peduncle minimum height	5.2–6	4.3–5.9
Caudal peduncle length	6.6–9	10.8–13.7
L 3° dorsal spine (longest) distance between pelvic fin bases	14.4–18.9	12–17.4
Meristic		
Dorsal fin rays	VII‐IX + 27–29^a^	VII + 27–30
Anal fin rays	I‐II + 29–30	I + 28–31
Pectoral fin rays	13–14	14–15
Ventral fin rays	9	9
Developed gillraker	1–2 + 8–10	1–2 + 8–11
Branchiostegal rays	7	–
Vertebrae	32	30–31
Scales in lateral line	66–72	73–82
Soft dorsal‐fin base scutes	25–28	27–29
Soft anal‐fin base scutes	27–30	27–30
Caudal fin rays	3–4 + 13 + 3–4	–

^a^ Nine spines in only one Moroccan specimen of 22.7 cm *L*_S_ (Furnestin et al., 1958).

#### 3.1.4 Species delimitation

**FIGURE 4:**
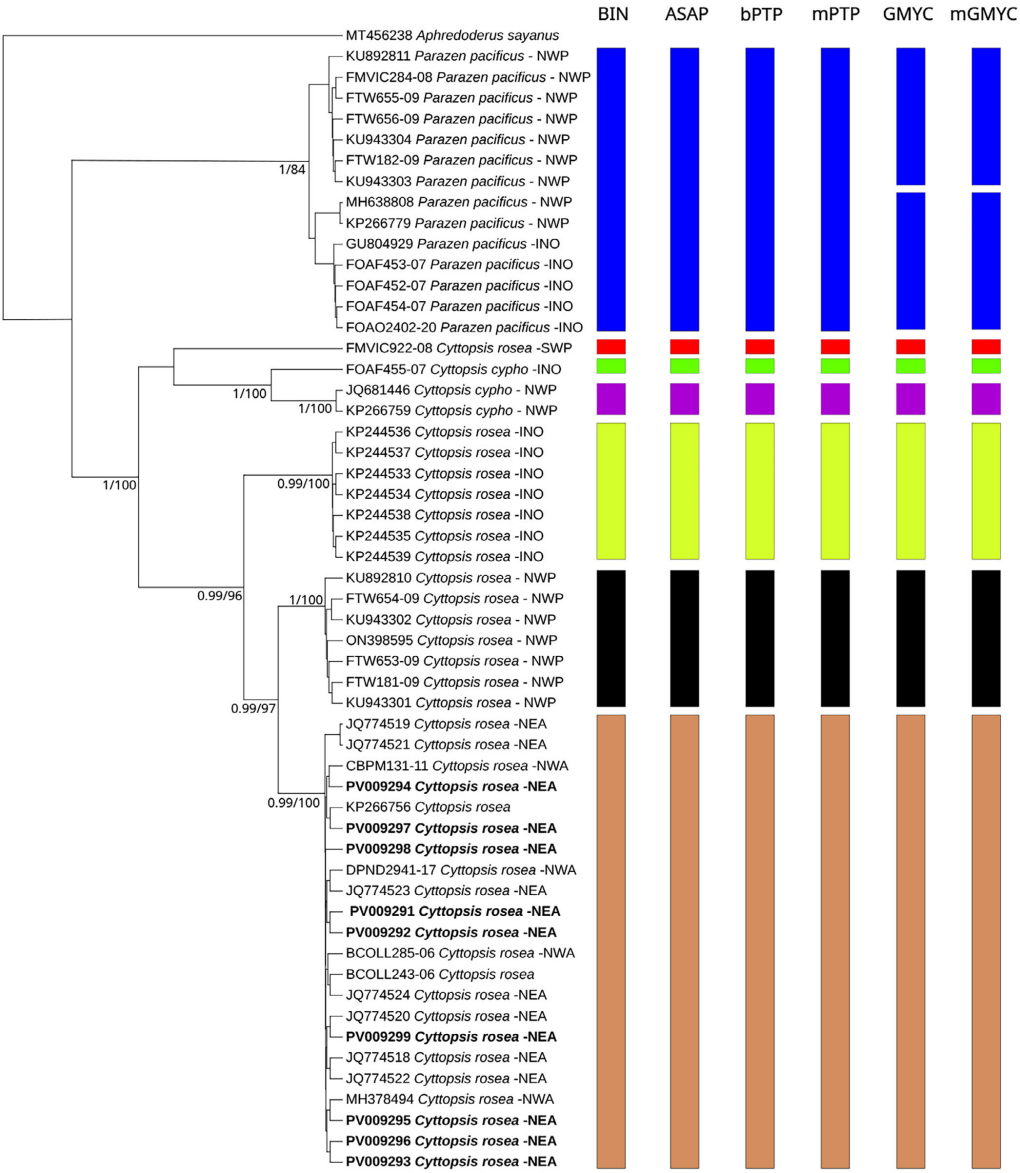
Consensus phylogenetic tree of 54 *COI* sequences belonging to the family Parazenidae plus one outgroup species (*Aphredoderus sayanus*). Node numbers represent posterior probability (>0.9) and bootstrap (>75). Sequences obtained in the present study are highlighted in bold. Each sequence includes its GenBank/Bold unique label, species identification and abbreviation of geographical origin: NWP, northwestern Pacific; SWP, southwestern Pacific; INO, Indian Ocean; NEA, northeastern Atlantic; NWA, northwestern Atlantic. The species delimitation results include distance‐based (Barcode Index Number [BIN] and Assemble Species by Automatic Partitioning [ASAP]) and tree‐based (Bayesian Poisson tree processes [bPTP], multi‐rate Poisson tree processes [mPTP], generalized mixed yule‐coalescent [GMYC] and multiple threshold generalized mixed yule‐coalescent [mGMYC]) methods.

In the resulting consensus phylogenetic tree, all sequences formed monophyletic groups based on their morphological identification with high statistical support in both posterior probability and bootstrap, with the exception of FMVIC922‐08 (*C. rosea*), from New Zealand, whose position in the tree remains unresolved (Figure 4). Species delimitation analyses showed a remarkable consistency among them. Four out of six, including distance (BIN, ASAP) and phylogenetic tree (bPTP, mPTP) methods, gave exactly the same results with a total of seven clades, while both GMYC analyses agreed on eight. For *C. rosea*, all delimitation analyses found the same three clusters in its monophyletic clade with remarkable geographic patterns. The first clade comprises seven sequences (KP244533‐39) from the Indian Ocean; the second includes another seven sequences from northwestern Pacific Ocean (China, Taiwan, Japan) while the largest clade contained 21 sequences representing northeastern and northwestern Atlantic Ocean and two sequences (KP266756, BCOLL243‐06) of unconfirmed origin (Figure 4). The three sequences belonging to *C. cypho* were divided in two clusters for all the delimitation analyses, the first with JQ681446 and KP266759 from the northwestern Pacific Ocean (South China) and the second with FOAF455‐07 from the Indian Ocean (Western Australia). The monophyletic group with Parazen *Parazen pacificus* (Kamohara, 1935) sequences was statistically well supported, but the two subgroups into which it is split were not. Four out of six delimitation analyses considered *P. pacificus* a single species, while both GMYC split it in two. The first included sequences from the northwestern Pacific Ocean, Taiwan (FTW655‐09 and FTW656‐09) and China (FMVIC284‐08), while the second comprised sequences from Indian Ocean, Western Australia (FOAF452‐07; FOAF454‐07), South Africa (GU804929) and South China (MH638808; KP266779).

### 3.2 Key to Parazenidae species

Adapted from Tyler et al. (2003) and Mizumachi et al. (2022); taxonomic characters of *C. rosea* limited to those of the Atlantic Ocean.

1a. Body elongated, body depth 2.2–3 times in *L*_S_; 8 dorsal‐fin spines; 7 pelvic‐fin rays; pelvic‐fin positioned about midway between pectoral‐fin base and anus; pectoral‐fin rays usually 16, rarely 15; procurrent caudal‐fin rays 7 or 8; scales spinoid; no large buckler scales along ventral midline; low sheath of scales along soft dorsal‐ and anal‐fin bases…………… Subfamily Parazeninae, *Parazen pacificus* (Kamohara, 1935).

1b. Body oval, body depth 1.3–2.5 times in *L*_S_; dorsal‐fin spines usually 6 or 7, rarely 8; pelvic‐fin rays 9; pelvic‐fin positioned under or slightly in front of pectoral‐fin base; pectoral‐fin rays usually 13 or 14, rarely 15; procurrent caudal‐fin rays 3 or 4; scales cycloid; large buckler scales along ventral midline; no sheath of scales along soft dorsal‐ and anal‐fin bases ………………………Subfamily Cyttopsinae (2).

2a. Dorsal‐fin spines usually 6, sometimes 7; pectoral‐fin rays usually 13, sometimes 14; large buckler scales along ventral midline of isthmus forming a continuous, sharp‐edged median spiny keel (nearly all buckler scales strongly overlapping); honeycomb pattern on bones on top of head visible externally…………………………… Genus *Stethopristes* Gilbert, 1905, *Stethopristes eos* (Gilbert, 1905).

2b. Dorsal‐fin spines usually 7, sometimes 8 or 9; pectoral‐fin rays usually 14, sometimes 13 or 15; large buckler scales along ventral midline of isthmus forming a partially separated spiny keel (a few bucklers not overlapping, especially first, on isthmus, and second, between pelvic‐fin bases); no honey comb pattern on bones on top of head, opercles, and lachrymal‐infraorbitals, only long ridges……………………………. Genus *Cyttopsis* Gill, 1862 (3).

3a. A faint dark lateral spot posteriorly on the body; 55–64 scales in lateral line; interspace between spines on 4th and 5th abdominal narrower; orbit diameter 13.6%–16.6% *L*_S_; interorbital width 5.8%–6.6% *L*_S_; snout length 21.1%–27.4% *L*_S_; mandible length 22.6%–25.6% …………………*Cyttopsis cypho* (Fowler, 1934).

3b. Without dark lateral spot posteriorly on the body; 66–72 scales in lateral line; interspace between spines on 4th and 5th abdominal scutes wide; orbit diameter 13%–15.9% *L*_S_; interorbital width 6.6–9.1% *L*_S_; snout length 16.3%–23.3% of *L*_S_; mandible length 22.1%–26.9% of *L*_S_………… *Cyttopsis rosea* (Lowe, 1843) Atlantic Ocean.

## 4 DISCUSSION

Fish taxonomy is far from complete. The taxonomic trend throughout the history of ichthyology has been to reduce the number of species within a given taxon through successive taxonomic revisions (synonymous species). As a result, many fish species are now widely distributed and morphological differences, when known, are attributed to natural intraspecific variation. In contrast, the use of molecular techniques seems to indicate the presence of a large hidden diversity and putative cryptic species with a more reduced distribution area (Bañón et al., 2024; Pavan‐Kumar et al., 2016).

Morphological and meristic characteristics of *C. rosea* specimens examined were consistent with previous descriptions of the species. However, differences were found between the Atlantic and Pacific specimens. The most remarkable are the caudal peduncle, which is longer in the Pacific than in the Atlantic specimens (10.8%–13.7% *L*_S_ vs. 6.6%–9% *L*_S_), the number of vertebrae (32 in the Atlantic vs. 30–31 in the Pacific) and the lower number of scales in the lateral line of the Atlantic (66–72) compared to the Pacific specimens (73–82). According to Heemstra (1980), the number of lateral line scales in *C. rosea* ranges from 73 to 84, in the same range as the 73–82 found in the Pacific specimens by Mizumachi et al. (2022). However, Heemstra's counts are based on the examination of six specimens from three different geographical areas, Japan, South Africa and the Northeast Atlantic (Heemstra, 1980), so it is not possible to establish geographical differences. Vaillant (1888) reported 53 scales in the lateral line of an Atlantic specimen. Although this number is anomalous and outside the known range of the species, it could support the lower number of scales in Atlantic specimens.

More striking is the presence of two small spines on the lower jaw. Although these spines are shown in radiographs and drawings of the species (Peters et al., 2024; Rosen, 1984; Tyler et al., 2003), to our knowledge this feature has only been recorded by Jordan and Fowler (1902) in the description of *C. itea*, a synonym of *C. rosea*. The taxonomic significance of this character needs to be reviewed.

Single‐gene species delimitation methods have been widely used for biodiversity studies (Ramirez et al., 2023). However, taking into account their different assumptions and principles, they can be affected differently by the data set features (Magoga et al., 2021). For instance, the barcoding gap estimation can vary due to a higher number of sampled haplotypes, which correlates with an increased probability of shared haplotypes between closely related species, or because of a mismatch between the number of intra‐ and interspecific sequences, therefore altering the results obtained through genetic distances (Phillips et al., 2019). Similarly, tree‐based methods rely on the upstream correct preparation of phylogenetic trees (Goulpeau et al., 2022). GMYC can overestimate the number of taxa, particularly in species with a strong intraspecific genetic structure (Hilário et al., 2021). However, approaches like GMYC and PTP are considered robust when properly used since they make stronger assumptions about speciation and distribution of genetic diversity because they use a speciation model and require tree‐building with an a priori model of nucleotide substitution (Hubert et al., 2024). Therefore, each delimitation tool has its own advantages and limitations, and by combining multiple delimitation algorithms results interpreted with the help of other sources (morphology, ecology, biogeography, etc.) limits the impacts of biased sampling or particular evolutionary histories (Carstens et al., 2013).

Our study revealed discrepancies between molecular taxonomy and morphological identification. The results indicate the presence of three distinct *C. rosea* lineages, suggesting the possibility of cryptic genetic differentiation and/or misidentification.

This taxonomic inconsistency was previously observed by Costa et al. (2012) for Atlantic specimens and Teramura et al. (2022) for Pacific specimens. These authors noted that *C. rosea* sequences in BOLD fall into different BINs, indicating divergent and overlapping lineages. Now we can say that these BINs are fully supported by our results, which combine several independent approaches. Therefore *C. rosea* is a complex of cryptic species, classified as a single species on the basis of morphological similarities, but with distinct genetic lineages.

Interestingly, *C. rosea* complex shows a clear geographic pattern, clustering together the samples from the Indian Ocean, the northwestern Pacific and the North Atlantic. Similar results for marine fish species with a supposed global distribution, which turned out to be several geographically distinct species, have been found in Shortfin neoscopelid *Neoscopelus microchir* Matsubara, 1943 (Bañón et al., 2024), Reef croaker *Odontoscion dentex* (Cuvier, 1830) (da Silva et al., 2023) or in the genus *Monacoa* Whitley, 1943 (Poulsen et al., 2016).

The morphological differences found in *C. rosea* between Atlantic and Pacific specimens may represent allopatric speciation between specimens with large geographical separations, as indicated by molecular analyses. According to these results, *C. rosea* forms a species complex consisting of at least three putative species. Based on the type locality, *C. rosea* would be an Atlantic species, while there may be up to two other morphologically similar Indo‐Pacific species that have not yet been described. The addition of a species key to the family Parazenidae and the geographical representation of the *C. rosea* molecular operational taxonomic units will be useful for the future delimitation of this species complex.

Despite being represented by only three sequences, all delimitation analyses showed two independent clusters for *C. cypho*. This is a remarkable result taking into account the low sample size for this species, which normally hides genetic diversity (Luo et al., 2015). Further analyses are needed to unravel the hidden diversity of *C. cypho*.

A particular case is the sequence morphologically identified as *C. rosea*, but which has no clear position in the tree and therefore is not part of the *C. rosea* complex. All delimitation analyses agreed to consider it as a separate species but the relationships among it, *C. cypho* and *C. rosea* complex remain unclear.

Similarly, previous taxonomic studies on *P. pacificus* have indicated the existence of cryptic species. Based on morphological characters, Kotlyar (2001) suggested that the Atlantic parazenid specimens of *P. pacificus* may be a separate species from the Indo‐Pacific specimens. Morphological differences and *COI* gene analysis from multiple specimens of *P. pacificus* across its geographic range support the hypothesis that this species may comprise at least two cryptic species, Atlantic and Indo‐Pacific (Corcoran & Grande, 2023; Singer et al., 2022). In our analyses, the lack of public sequences from the Atlantic Ocean did not allow us to test this hypothesis. However, while distance‐based methods and PTP both identified *P. pacificus* as a single species, GMYC split it into two groups (northwestern Pacific vs. northwestern Pacific and Indian Ocean). These results should be interpreted with caution because PTP is generally regarded as providing more accurate species delimitations than GMYC, which has a tendency to overestimate species numbers (Tang et al., 2014). Further analysis including samples from the North Atlantic will be necessary to resolve the taxonomic status of *P. pacificus*.

Discrepancies between morphological identification and molecular taxonomy are not uncommon in other zeiform fishes such as John Dory *Zeus faber* L. 1758 (Ward et al., 2008), silvery John Dory (Lowe, 1852) *Zenopsis conchifer* (Matusevich et al., 2024) or mirror Dory *Zenopsis nebulosa* (Temminck & Schlegel, 1845) (Wang et al., 2012), suggesting hidden diversity within each nominal species. It is also not uncommon for new zeiform species to be described, such as *Zenopsis stabilispinosa* Nakabo, Bray & Yamada, 2006 or *Zenopsis filamentosa* Kai & Tashiro, 2019. All these indications suggest that the taxonomy of zeiforms is far from complete, pending the description of new cryptic species.

The growing number of cryptic fish species indicates that traditional morphological techniques may no longer be adequate for effective species discrimination. However, some species currently classified as cryptic might, on closer morphological and spatial analysis, be reclassified as pseudocryptic (Lajus et al., 2015). Really, the diagnostic characteristics of many fishes, mainly non‐commercial or deep‐sea fishes, are often based on ancient manuscripts by examining only a few specimens, and these descriptions remain valid to this day (Bañón et al., 2022). The lack of fish taxonomists, the difficulty of examining specimens from different geographical areas and the rejection of descriptive work by many scientific journals are behind these gaps in knowledge. The resulting lack of understanding of intraspecific variation complicates the identification of species boundaries, hindering accurate assessments of species diversity and distribution (Frutos et al., 2022).

The challenge for the future is not only to identify fish species with hidden biodiversity, but also to describe these new species. Unfortunately, all indications are that this will be a slow process given the lack of trained taxonomists and institutional support for biodiversity research.

## AUTHOR CONTRIBUTIONS

R.B. conceived the idea and conducted morphological analyses. D.B.G., F.B., A.S.C. and A.d.C. conducted sequencing and molecular analyses. F.B. conducted the biogeography representation. F.B. and J.C.A. compiled the specimens. J.D.B.V. made the X‐ray images. R.B. wrote the first draft of the manuscript, which was further elaborated by D.B.G., F.B. and A.d.C. All authors critically reviewed and approved the manuscript.

## FUNDING INFORMATION

This research was developed thanks to the European Maritime, Fisheries and Aquaculture Fund (PORCUDEM‐20233FMP001). This research was funded by national funds through the Fundação para a Ciência e a Tecnologia within the scope of the Strategic Funding UIDB/04423/2020 (https://doi.org/10.54499/UIDB/04423/2020), UIDP/04423/2020 (https://doi.org/10.54499/UIDP/04423/2020), and LA/P/0101/2020. (https://doi.org/10.54499/LA/P/0101/2020). A.d.C. research was partly funded through a contract for competitive reference research groups of the Xunta de Galicia (ED431C 2019/28).

## CONFLICT OF INTEREST STATEMENT

The authors declare that they have no conflicts of interest.
